# When the Sound Becomes the Goal. 4E Cognition and Teleomusicality in Early Infancy

**DOI:** 10.3389/fpsyg.2017.01585

**Published:** 2017-09-25

**Authors:** Andrea Schiavio, Dylan van der Schyff, Silke Kruse-Weber, Renee Timmers

**Affiliations:** ^1^Institute for Music Education, University of Music and Performing Arts Graz, Austria; ^2^Department of Music, University of Sheffield Sheffield, United Kingdom; ^3^Centre for Systematic Musicology, University of Graz Graz, Austria; ^4^Faculty of Education, Simon Fraser University Burnaby, BC, Canada; ^5^Faculty of Music, University of Oxford Oxford, United Kingdom

**Keywords:** music cognition, musical development, exploratory behaviors, music in infancy, embodied cognition, embodied music cognition, 4E cognition

## Abstract

In this paper we explore early musical behaviors through the lenses of the recently emerged “4E” approach to mind, which sees cognitive processes as Embodied, Embedded, Enacted, and Extended. In doing so, we draw from a range of interdisciplinary research, engaging in critical and constructive discussions with both new findings and existing positions. In particular, we refer to observational research by French pedagogue and psychologist François Delalande, who examined infants' first “sound discoveries” and individuated three different musical “conducts” inspired by the “phases of the game” originally postulated by Piaget. Elaborating on such ideas we introduce the notion of “teleomusicality,” which describes the goal-directed behaviors infants adopt to explore and play with sounds. This is distinguished from the developmentally earlier “protomusicality,” which is based on music-like utterances, movements, and emotionally relevant interactions (e.g., with primary caregivers) that do not entail a primary focus on sound itself. The development from protomusicality to teleomusicality is discussed in terms of an “attentive shift” that occurs between 6 and 10 months of age. This forms the basis of a conceptual framework for early musical development that emphasizes the emergence of exploratory, goal-directed (i.e., sound-oriented), and self-organized musical actions in infancy. In line with this, we provide a preliminary taxonomy of teleomusical processes discussing “Original Teleomusical Acts” (OTAs) and “Constituted Teleomusical Acts” (CTAs). We argue that while OTAs can be easily witnessed in infants' exploratory behaviors, CTAs involve the mastery of more specific and complex goal-directed chains of actions central to musical activity.

## Introduction

Elysa is a healthy 6-month old human infant. Today her caregiver brings her a new toy, a colorful rattle. He shows it to her and places it in front of her. This excites Elysa, as we can infer from her body motions and facial expressions. As a consequence of this state of excitement, she moves and reaches for it. But as soon as she touches the rattle, something unexpected happens: it produces a sound. While Elysa initially appears to be surprised, she also seems to enjoy the experience and keeps moving the rattle, making even more sounds. By engaging in such activities (interacting with the toy, looking at it, and listening to its sounds) over days and weeks, Elysa improves her ability to make controlled and motivated engagements with the rattle. She is learning bodily awareness and control and, in the process, develops important understandings of how perceptual and proprioceptive modalities (movement, touch, sight, and sound) correspond with each other. Through this form of multisensory exploration, where perception and action are continuously coupled, Elysa will discover the causal relationships between her actions and the auditory feedback coming from the toy.

The exploratory behaviors described above are commonly observable in infants of this age (see Pierroutsakos and DeLoache, 2003; Sheya and Smith, 2010a,b; Soska et al., 2010). They reach for, grasp, and manipulate objects in their environment as soon as they develop adequate motor skills. In doing so, they also encounter the *sound properties* of the objects at hand, which may offer further opportunities for exploration and for developing new types of actions^1^. Through such modes of discovery, infants develop patterns or “repertoires” of actions that are—among other things—specifically directed toward sound-making goals (Clarke, 2005; Delalande, 2009). In this paper, we consider how a closer examination of such exploratory activities may reveal new ways of understanding musical experience in early infancy. More specifically, we suggest that the ontogenetic emergence of human musicality may be best understood by referring to the *unique, adaptive, self-organizing*, and *creative* ways infants explore and interact with the possibilities-for-action afforded by their (sonic) environment (see Gibson and Walker, 1984). Where more traditional views focus on describing how pre-given genetic developmental “programs” respond to environmental stimuli, our approach aims to provide a preliminary taxonomy of the general kinds of music-related activities that emerge in infancy through active processes of corporeal engagement with the world. In doing so, we will make a general distinction between *protomusical* behaviors, which include music-like utterances and movements that do not entail a primary focus on sound itself (e.g., emotional-affective interactions with the caregiver; see e.g., Dissanayake, 2000; Trevarthen and Malloch, 2000; Cross and Morley, 2009), and *teleomusical*^2^ behaviors, which describe the goal-directed actions infants adopt to interact with the sound-properties of their environment. This distinction, we argue, will help us capture a number of new features of early musicality that may be relevant for future empirical and theoretical work in the field of developmental music psychology, and music cognition more generally. Additionally, we will also discuss how this perspective may be further developed in association with the so-called “4E” approach to mind—which conceives of cognition as an Embodied, Embedded, Enacted, and Extended phenomenon (see Rowlands, 2010). The juxtaposition of these four dimensions may offer a useful framework for studying the emergence and development of musical behaviors in terms of the action-perception cycles that characterize cognition in infancy and beyond (Thelen and Smith, 1994).

Before we start, we should note that similar insights have already been addressed in music scholarship (e.g., Clarke, 2005; Reybrouck, 2005, 2012; Leman, 2007; Leman et al., 2009; Krueger, 2013; Addessi, 2014, 2015; Leman and Maes, 2015; Schiavio and Altenmüller, 2015; Schiavio and van der Schyff, 2016; Schiavio et al., 2016). And indeed, the orientation we develop here has antecedents in—and is inspired by—the work of Piaget (1952, 1964); Imberty (1995, 2009), and Delalande (1984, 1998, 2009, 2013) among others. It also aligns with more recent research on action-perception coupling and mirror neurons in music education (e.g., Addessi, 2012; Schiavio and Timmers, 2016); embodied cognition and music education (e.g., Volpe et al., 2012; Addessi et al., 2015; Nijs and Leman, 2016; van der Schyff et al., 2016); infant-caregiver vocal interaction (e.g., Gratier, 2003, 2007; Nakata and Trehub, 2004; Gratier and Trevarthen, 2008); musical parenting and music education (e.g., Papoušek, 1996; Ilari et al., 2011); and the role of action experience for musical development (e.g., Philips-Silver and Trainor, 2007; Gerson et al., 2015b). Drawing on this body of work we aim to (i) develop new bridges between disciplines such as music education, developmental psychology, cognitive neuroscience, theoretical biology, and philosophy of mind, (ii) extend previous findings within such interdisciplinary scholarship, and (iii) offer more nuanced ways of discussing how musicality develops in infancy. Moreover, to the best of our knowledge, a systematic investigation aimed at exploring music in infancy through the lenses of the 4E approach is still missing. Therefore, this paper also aims to begin to fill this gap and to contribute to current state-of-the-art in early music cognition research more generally.

## The mutuality of action and perception

In this section we would like to introduce an approach to musical development not based in pre-given (or genetic) programs, but rather in the deep continuity between action and perception. In doing so, we will explore research in neuroscience and developmental studies that reveals the centrality of situated, agentic, goal-based action for the acquisition of skill and understanding, and show how this work is relevant in musical contexts. We begin by considering contributions involving auditory perception and cognition in infancy, arguing that such capacities should be understood in terms of the ways infants actively *explore* and *make sense* of the environments they inhabit. In support of this, we then discuss work on the Mirror Neuron System (MNS) that illuminates the action-perception cycles inherent to understanding and engaging in goal-directed activity more generally. Here we make some preliminary suggestions for what such insights might mean for musical development. These musical concerns will be developed in more detail in the section What is *Teleomusicality*? Before that, however, let us go back to Elysa.

### Sonic experience in action

Elysa's world presents regularities and novelties that stimulate her curiosity, affording a number of emerging activities that allow (further) possibilities for exploration. And indeed, sonic events represent some of the first and most important spontaneous ways young infants engage with and make sense of the world (see Malloch and Trevarthen, 2009; Countryman et al., 2016). Unlike vision, the auditory system is already highly functional at birth (Bredberg, 1968; see also Lecanuet et al., 1986, 1988, 1991, for discussion on pre-natal sounds experiences), and infants appear to display great acuity to sounds (see Trehub, 2009; Perani et al., 2010; cf. Keefe et al., 1994). Evidence from scalp-recorded auditory evoked potentials suggests that fetuses are already sensitive to auditory events, although amniotic fluid and maternal tissues may limit their experience (Smith et al., 2003). According to Moore and Jeffery (1994), while hearing starts at the 22nd week of gestation, both cochlea and central auditory pathway are still immature (structurally and functionally). It is suggested, then, that it is at 5–6 months (postnatal) of age that frequency and temporal resolution can be understood to have matured properly (e.g., Werner, 2002). 5-month-old infants, for example, are able to discriminate differences in frequency of less than one half step (Olsho, 1984). And starting from 8 months of age they begin to make pitch discriminations based on an awareness of melodic contour (Trehub et al., 1984). Interestingly, infants are also found to be better than adults at discriminating between certain typical aspects of Western music (e.g., a pitch variation; see Trainor and Trehub, 1992). While adults are easily able to detect changes that violate expectations associated with Western musical structures (such as non-diatonic variations), they have more difficulties with diatonic changes—that is, with pitch alterations that do not deviate from the tonal structure typical of Western classical music. Infants, on the other hand, “detected both changes equally well” (Trainor and Trehub, 1992, p. 399). Similar results have also emerged in rhythm perception studies, where it has been found that, when compared to adults, infants display greater accuracy in detecting changes in newly listened rhythmical patterns—seemingly independently of any familiarity they may have with the rhythmical structures typical of their own culture (Hannon and Trehub, 2005). However, a preference for rhythmical structures common to the infant's own culture emerges later (Soley and Hannon, 2010). Other studies suggest that young infants also discriminate between consonant and dissonant sounds, as well as sound properties such as location, duration, and pitch (see also Trehub, 2003a,b,c,d,e).

This research reveals that infants have the ability to attend to sounds and music in very nuanced ways (which appear to become narrowed over time through factors such as enculturation). At a very young age, Elysa is already able to capture many features of the auditory events in her environment. Her sensitivity to melodic, rhythmic, and harmonic^3^ structures is stunning. However, as with other human infants, she is not just a *passive* receiver of sonic inputs; her experiences with sounds do not simply involve *responses* to given auditory stimuli. Infants like Elysa, rather, may be observed to spontaneously and deliberately *generate* music-like patterns, developing new rhythmic or melodic structures (Imberty, 1995; Papoušek, 1996). Often this occurs through vocal and rhythmic interactions with the caregiver that involve bodily movement (Condon and Sander, 1974; Jaffe et al., 2001; Custodero and Johnson-Green, 2003, 2008; Malloch and Trevarthen, 2009). However, the musical world of an infant also includes more independent processes of exploration and discovery that involve the development of action understanding and prediction. With regard to this point we should note here that although infants' vocal productions are important constituents of their musical development (see Stern et al., 1975, 1982; Gratier, 2003; Addessi, 2008a,b; Eckerdal and Merker, 2009), these also display a different phenomenology from manual explorations. As we cannot provide here an extensive overview of both, we will mainly focus on the latter for the rest of the paper. Moreover, while some of the communicative features attributed to vocal sounds are also inherent to manual skills, many of the goals afforded by sensorimotor explorations (e.g., playing with a ball, grasping a toy, etc.) remain unachievable in terms of vocal production (infants cannot vocally manipulate their environment directly—although they can certainly vocally “ask” for something to be done). This said, in the following section we develop possibilities for understanding the development of sensorimotor knowledge from a neural perspective. Here we consider the role two classes of motor neurons play in developing repertoires of goal directed activity that are relevant to an agent's lived experience. This will help to inform the discussion of musical development that follows.

### Mirroring and understanding

A basic form of action understanding is observed in newborns (see Craighero et al., 2011). At around 6 months of age infants begin to employ controlled grasping and appropriate pre-configurations of the fingers (von Hofsten, 1982). This develops rapidly into more directed reaching and grasping behavior. Interestingly, the development of these more sophisticated behaviors appears to coincide with the emergence of the infant's ability to predict the goals of the actions of other people involved in similar activities (i.e., reaching for objects; Woodward, 2009). This helps reveal the *bidirectiona*l way action and perception influence each other (Thelen, 1989, 1994; Thelen et al., 2001; Gerson and Woodward, 2014a,b; Gerson et al., 2015a). As Kanakogi and Itakura (2011) suggest, “the developmental onset of infants' ability to understand an action, reflected by the ability to predict the goal of others' action, is synchronized with the developmental onset of their own ability to perform that action, and […] there is developmental correspondence relationship between the ability to predict the goal of an action and the ability to perform that same motor action” (see also Gergely et al., 1995; van der Meer et al., 1995; Woodward, 1998; Daum et al., 2011; Cannon et al., 2012, 2016; Robson and Kuhlmeier, 2016). With this in mind, we provide next a conceptual review of the main findings concerning Mirror and Canonical neurons, and consider how this research might align with and support the action-oriented, and goal-based, approach to early musical development outlined in the following sections.

Mirror Neurons are sensorimotor neurons that become active both when performing a motor action and when observing or hearing a similar action made by another individual (Di Pellegrino et al., 1992; Gallese et al., 1996; Kohler et al., 2002). They were first discovered in the ventral premotor cortex of macaque monkeys, and were subsequently observed in the brains of surgical patients (Mukamel et al., 2010). A common way to assess mirror-like activity in the brain involves the use of EEG. A number of studies with adults and infants (e.g., Cochin et al., 1998, 1999; Nyström, 2008) have confirmed mirror-like activity by reporting *mu rhythm* desynchronization during both production and observation of actions. In terms of fMRI studies, a meta-analysis conducted by Molenberghs et al. (2012) appears to locate mirror-like activity in inferior frontal gyrus, ventral premotor cortex, IPL, primary visual cortex, and cerebellum, among other areas (see Keysers and Gazzola, 2010). In addition to these Mirror Neurons, another set of neurons, dubbed Canonical Neurons, discharge when we observe a graspable *object* without performing any movement, as well as when we grasp that object (Rizzolatti et al., 1988). In a well-known study, Fadiga et al. (1995) show that the motor evoked potential of the human motor cortex increases during simple observation tasks, reflecting the muscle activity relevant for the actual performance of the observed motor behavior.

Put simply, Canonical Neurons discharge during the execution of a motor act, and in response to the presentation of an object in the observer's peripersonal space. Typically, they display congruence between the coded action (e.g., grasping) and the physical properties of the observed object (e.g., a small ball)^4^. Mirror Neurons, instead, fire both during the performance of an object-directed action and when that same action is observed in others. They are elicited not by the precise movements performed but, rather, by the *goal* of the given action (Rizzolatti and Sinigaglia, 2008). In other words, what really matters for these neurons is not the kinematics (e.g., contractions of single groups of muscles), but the *goal-directedness* involved in grasping as such (see Fogassi et al., 2005; Keysers, 2007). Mirror-like activations are elicited by those actions the observer knows how to perform—those actions that are in his or her “motor repertoire”^5^.

An elegant fMRI experiment carried by Buccino et al. (2004) provides further clarification regarding this point. They asked participants to watch two groups of silent videos wherein a man, a monkey, and a dog perform (i) ingestive actions and (ii) communicative actions. The actions in the first set of videos involved *biting food*, and all three animals were engaged in the same action. In the second set of videos, however, the communicative motor behaviors were different—the observed actions were relevant to each species, such as *talking* (humans), *lip smacking* (monkeys), and *barking* (dogs). The results of the experiment showed that for the first condition (biting) there was a clear overlapping of the brain areas that became active during the observation of the videos. Indeed, biting is a relevant aspect of the motor activity of all three animals. And thus, “watching the three videos produced the activation of two sites (a rostral and a caudal) in the inferior parietal lobule as well as the posterior part of the inferior frontal gyrus and the adjacent precentral gyrus” (Rizzolatti and Sinigaglia, 2008, p. 132). However, the results of the second condition (communication) were significantly different. The mirror-like activation was much weaker when the human participants observed acts like monkey lip smacking, and disappeared when they watched the dog barking.

Finally, a well-known study by Kohler et al. (2002) showed that a subclass of F5 mirror neurons fire not only when a monkey observes or performs a given action, but also when it hears the sound produced by the action itself. A goal-directed action, therefore, can be understood independently from the format of the sensory information. The (sonic) motor acts employed in this experiment involved breaking a peanut and ripping apart a sheet of paper—these are acts that are relevant for the monkey, and are thus present in their acquired motor knowledge. As such, we could infer that when the monkey heard only the sound of a known action, it automatically activated in its brain the motor plan necessary to perform the very same action (see also Damasio, 2003; Gallese, 2005; for work related to music, readers may consider Bangert and Altenmüller, 2003; D'Ausilio et al., 2006; D'Ausilio, 2007, 2009; Overy and Molnar-Szakacs, 2009). Such multi-modal forms of action understanding, therefore, are not wholly pre-given, but rather develop via the animal's history of embodied engagement with the socio-material environment. As we will consider next, these insights may help us better understand how as infants develop richer motor repertoires, they also gain the ability to understand the actions of others, as well as the capacity to enact new forms of goal-directed behavior through exploratory forms of action-as-perception.

### From action to musical experience

Human infants are active explorers of their physical and social world (McCall, 1974; Ruff, 1984, 1986). From birth they develop sets of multimodal skills, and engage in meaningful interactions with persons and objects in their environment, gaining relevant experience and knowledge of its regularities and possibilities for action. As we have begun to discuss, it is argued that such interactions entail a form of bidirectional dependency, where the young organisms' developmental trajectories do not simply rely on internal instructions “programmed” to respond to environmental stimuli (see Bertenthal and Campos, 1987; Gottlieb, 1991a,b). Rather, bio-cognitive development involves active processes of organism-world interactivity. This echoes Gibson's (1979) famous assertion that “[w]e must perceive in order to move, but we must also move in order to perceive” (p. 223), pointing to the mutual influence of perception and action (see also Merleau-Ponty, 1945).

The insight that motor and perceptual processes are dynamically determined by each other has allowed scholars to go beyond traditional approaches to the study of human action. The latter were mostly interested in discovering the “motor programs” of the central nervous system, and in understanding how such programs might generate behavioral outcomes. In contrast to this, we may consider the now-classic work by Esther Thelen and her collaborators (see e.g., Thelen, 1989, 1994, 2000; Thelen and Smith, 1994; Thelen et al., 2001; Smith and Thelen, 2003; see also Spencer et al., 2006), which offers an impressive collection of contributions aimed at describing motor development in terms of the “complex and ongoing interplay of arousal, attention, motivation, biomechanics, neuro-motor control, muscle performance, head-arm-trunk posture, and experience” (Galloway, 2005, p. 105).

For example, one might think of the developmental transition from one mode of exploratory action, such as crawling, to another, such as walking (see Oudgenoeg-Paz et al., 2012; He et al., 2015). Here we may return to Elysa: she is now 10 months old and by now has been crawling for some time. However, she has just developed enough coordination and physical strength to begin to walk upright. This involves a “shift” in her behavior, where the pattern of crawling—which was stable for months—is now *destabilized* by the pattern of standing and walking. As Smith and Thelen comment: “there is no ‘program’ for crawling assembled in the genes or wired in the nervous system. It self-organizes as a solution to a problem (move across the room), later to be replaced by a more efficient solution. Development is a series of evolving and dissolving patterns of varying dynamic stability rather than an inevitable march toward maturity” (Smith and Thelen, 2003, p. 344). The following passage can further clarify this idea:

A baby is provided by nature with some very helpful equipment to start its long course of learning about and interacting with the world. A baby is provided with an urge to use its perceptual system to explore the world; and it is impelled to direct attention outward toward events, objects and their properties, and the layout of the environment (Gibson, 1988, p. 7; quoted in Thelen and Smith, 1994, p. 314).

Infants seek out information for their senses; they actively engage with the properties of the environment to assess and make sense of the possibilities it offers. Our understanding of music should thus take into consideration this biological disposition that young infants display. With regard to this point, we could perhaps try to propose a very general definition of “music” in early infancy. We might conceive of music as an *emerging property of the ongoing relation between brain, body, and environment*; a property that can only emerge when infants actively engage in motivated exploratory behaviors involving sound-related outcomes. There can be “musical activities” when those sound-properties are explored and engaged with in creative ways—that is, when the goal of the exploration becomes the sound per se^6^. Concerning this last point, Delalande (e.g., 2015) draws on the phenomenological tradition proposed by Schaeffer (1966; see also Chion, 1983, 1988; Nattiez, 1987) where the precise distinction between sound, noise, and music is revealed to be culturally influenced, and where focused explorations of sound may be better understood in terms of the complex acoustic (e.g., *timbral*) affordances of the “objet sonore” that arise as the infant engages with it. This last point resonates closely with our perspective. And indeed, we suggest that our focus on goal directedness and the development and re-enactment of specific repertoires of actions might help to extend such insights. By recognizing the deep continuity between culture and (motor) cognition, we maintain, like Delalande, that “music” cannot be reduced to an objective event, nor to a pre-given behavioral program. As such, we advocate for a dynamic framework—developed in continuity with interdisciplinary scholarship—that sees the motor components of action, perception, and cognition, as deeply fundamental for musicality to occur. As we will discuss further, this may help to expand Delalande's (2013) concept of the “conduite d'écoute” (conduct of listening), where he argues that music should not be first considered as an ensemble of stimuli (sonic or graphic), but rather as a bundle of conducts (actions) involved with making it and listening to it (p. 158). From this perspective, musical experience depends on the agency of the person who engages with it. It involves the adaptive capacities of the listener/player to create and enact meaningful relationships with the “objet sonore” (p. 164)—a process that involves the integration of emotional-affective and motor aspects (p. 230; see also Clarke, 2005, for similar points discussed within an ecological framework).

### Musical goals

Consider again our 10 month-old Elysa. At this age she has realized that sounds are dependent on the motor behaviors employed to produce them. However, she will not simply use the same chain of acts every time she explores the environment. That would be boring. By engaging in new or enhanced patterns of motor action, and by exploring different sounding objects, Elysa is able to experience, adapt to, and develop the sounding properties of her environment in richer ways and thus *enact* a range of meaningful relationships between them. Indeed, the more she familiarizes herself with certain actions (e.g., squeezing, shaking), the more she will be able to shift her attention toward other things, such as the visual and sonic properties of the objects she is engaging with. Delalande (2009) observes that when this happens (i.e., when the infant's attention is captured by the produced *sounds*, rather than only by her own action) the infant will start to play with sounds in a *meaningful* way. That is, Elysa may begin to apply some basic rhythmic or even melodic variations to her sound productions.

This kind of behavior seems to emerge properly between 6 and 10 months of age. Before 6 months of age, when objects that constitute goals for given actions (e.g., something to be grasped) appear in the infant's visual field, they do not pay attention to the physical properties of the object and instead concentrate on the action itself. However, by 10 months infants focus on both actions and their goals—e.g., objects and their properties. This resonates with a study by Ambrosini et al. (2013), who compared the anticipatory gaze of 6, 8, and 10 month-old infants (and a control group of adults) during goal-directed observational tasks. The researchers found a significant correlation between the ability to perform a given action and the capacity to successfully predict the goal of an observed action. This was noted in particular when 6-month old infants observed actions involving the precision grasping of objects (i.e., using thumb + index), which they are still not able to perform. Here they tended to focus on the action and not on what was grasped (they were not able to predict the goal of the action). By contrast, full-hand grasping is a well-established skill in the motor repertoire of most infants of this age. And indeed, when observing these types of action infants of this age did appear to predict with more precision the appropriate goal of the given grasping action. A related finding involves the relationship between age and the degree of gaze proactivity. Here advantages for goal prediction were found from 8 months onwards, with 10 month-old subjects showing faster gaze proactivity to precision grasping. Taken together, these results suggest that the ability to perceive and/or predict the goal of observed motor behaviors is action-specific—it depends on whether the infant is capable of actually carrying out the action observed.

This point is fundamental for our proposal. Infants develop repertoires of goal-directed actions that will allow them to explore the environment in a meaningful (e.g., musical) way. But, by mastering certain actions, infants also develop adequate perceptual abilities that could motivate further ways of interacting with the world. For example, by understanding the goal of a given sound-related activity performed by another individual (e.g., the caregiver, or a peer), infants could respond in creative ways, playing with sounds in a condition of mutual understanding (as long as both individuals have developed their motor vocabulary of actions). Here, a number of possibilities for empirical corroboration might be considered. For example, one could test whether infants who have developed an adequate repertoire of musically relevant actions are keener to play with others in musical games, when compared to infants who have less familiarity with musical actions. Or, we could investigate *mu* rhythm desynchronization in infants (with EEG) during the auditory perception of sounds produced by simple actions between two groups (similarly to the previously proposed possible study): infants who are familiar with repertoires of actions directed toward sound-making, and infants who are not. Given what we have considered thus far, it is predicted that *mu* rhythm desynchronization will be observed only in the first group, reflecting the activation of the mirror system (see e.g., Woodward and Gerson, 2014; Gerson et al., 2015a; Fox et al., 2016). Possible follow-ups might include the examination of perceptual and emotional responses to musical stimuli, and the presentation of musically affordative environments (i.e., toy musical instruments), between the same two groups.

At this point, it is important to briefly address the key notion of “musical goal.” This can be defined as the (more or less predicted) outcome of a musically motivated action. That is, the action involves engaging with sounds in a way that is not focussed on other primary interests (e.g., seeking attention from a caregiver etc.), but rather is chiefly concerned with the manipulation of sound itself^7^. This allows us to distinguish between (i) sound-related activities that are motivated by a genuine intention to play with sounds and (ii) sound-related activities that are focused on other goals. Good examples of (i) involve an infant hitting (“drumming”) a surface and waiting for a caregiver to do it too (preferably in different, novel, ways), or manipulating a sound-making object to get a desired result. Conversely, situations associated with (ii) could emerge when infants use sound to seek for or get the attention of their caregiver because they need nutrition, affection, or some other form care or interaction. In what follows, we shall name the former activity *teleomusicality*, and the latter *protomusicality*. Let us begin with *teleomusicality*.

## What is teleomusicality?

Between 6 and 10 months of age, infants develop the ability to integrate relevant information concerning action and objects in increasingly sophisticated ways (Sommerville et al., 2005). This can be understood in terms of a history of exploratory interactivity with an environment, where cycles of action-as-perception lead to even richer repertories of goal-directed acts. In the course of such processes, objects emerge as rich informational structures whose properties specify possibilities for action (i.e., affordances) in relation to the developing motor expertise of the infant (Perone and Oakes, 2006; Perone et al., 2009). But what happens when the goal of an action becomes a sound? That is, what happens when the goals of one's actions are not simply directed toward reaching for and moving an object, but rather are focused on the objects' sound properties? And what strategies might an infant employ to explore and manipulate the sonic environment created through her interactions with a sounding object?

Imberty (1995) suggests that the first musical productions of infants involve two key features, namely, *pivot* and *colmatage*. “Pivot” refers to a stable musical element, and “colmatage” specifies the variable nuances intrinsic to the musical event. However, both elements may arguably emerge only when an “attentive shift” has occurred—as we started to discuss in the section Musical Goals. This involves a shift in focus toward the *sonic* goals of the infant's actions, rather than (or in addition to) their kinematic and visual dimensions. To be clear, this does not mean that before the shift occurs infants are unable to perceive the sounds associated with musical events. Indeed, we have already discussed their considerable perceptual aptitude for sound. Rather, before this time, it appears that they are not able to focus on the sound as the *primary object of their action-as-perception*. In other words, the “attentive shift” permits the constitution of a first *musical context* where the infant's goal is intrinsically “musical.” We refer to this basic form of musical activity as *teleomusicality* (in Greek $\tau \overset{´}{\varepsilon }\lambda o\zeta$ means essentially “goal” or “result”). Likewise, we define the basic motor actions directed toward a musical goal as “teleomusical acts.” Here we may develop an approximate distinction between *original* and *constituted* teleomusical acts.

### Original teleomusical acts (OTAs)

These involve patterns of motor behavior that emerge in early infancy, such as grasping and shaking (e.g., a rattle placed in the hand). These actions are executed more or less spontaneously, enabling the basic forms of action-perception looping required to explore and engage with the *sonic* environment in which the infant is embedded. However, it is only by around 6 months of age that such activities may begin to be understood as properly *teleomusical*—it is at this age that infants focus specifically on the sonic properties of the objects they engage with (as opposed to the kinematic dimensions that seem to occupy them earlier on), and to begin to develop controlled actions in order to achieve sonic goals.

### Constituted teleomusical acts (CTAs)

These are not “primary” acts: they are built through the unification of sets of OTAs. Yet they rapidly develop kinematic fluidity, allowing the infant to execute them as unitary goal-directed actions. For adults, consider actions like playing a chord on the piano: this requires temporal coordination, sensibility of the fingers, wrists, arms, and back, expressivity, and so on. However, a skilled pianist does not integrate these categories one by one. Rather, she would achieve the goal (playing this chord in a certain way) through a fluid, non-reducible (holistic) execution, where the primary focus remains on the musical sound being produced, rather than on the kinematics behind it. For infants, similar behaviors may be witnessed when they hold a stick to hit another object and play with its sounds: after they gain some familiarity with the situation they are involved in, their attention—as with adults—is not focussed solely on the movement (kinematics), nor on the stick in its causal relationship with the drum, but mostly on the resulting sound.

### OTAs, CTAs and the three conducts

OTAs are basic and simple to perform. They begin as spontaneous proclivities for movement—they emerge in infancy seemingly without any imposition or help from the caregiver. As such, they may be understood as *self-organizing* behaviors (more on this shortly). They are also *plastic* and can be easily refined and performed in different contexts (through different strategies and motivations). Indeed, they are ecologically relevant with regard to how sonic affordances develop in a given subject-object relationship. Because of this, they also quickly become goal-directed toward the the properties of the sound and the enactment of protomusical structures. Every healthy infant possesses the skills necessary to perform the basic acts of hitting, plucking, or scratching (see also Godøy, 1997, 2003; Delalande, 2009). Accordingly, musical development in infancy could be considered as an emerging shift from the basic and spontaneous acquisition of OTAs to the development of CTAs. It is reasonable to argue that once a repertoire of CTAs is established, the young “musician” may then begin integrate other qualities into her musical life (such as extra-musical values). Indeed, she may now engage in musical activities that involve more complex forms of social co-ordination (such as performing together) and to develop the affordances of sounding objects in new ways through composition and improvisation. In line with this, we may now consider the three types of musical “conduct”^8^ offered by Delalande (2009), each of which is an extrapolation on the developmental framework Piaget (1964)^9^ refers to as “the phases of the game”:

1. *The explorative conduct* is based on the discovery of sounds and noises. It corresponds to the *sensorimotor game*, which, according to Piaget (1964) dominates the first 2 years of life. After 6 months from birth, as we discussed, the infant begins to explore the *auditory possibilities* of the surrounding environment in an increasingly controlled way. At this stage we suggest that OTAs are being developed into CTAs.
2. *The expressive conduct* corresponds to the phase Piaget's defined as *symbolic game*, and characterizes the years of kindergarten. Delalande suggests that during this period the child begins to attribute extra-musical values to sounds in association with certain situations, places, social roles, expectations, and so forth. This enriches the primary form of sensorimotor understanding with a broader domain of meaning attribution. This phase, then, may be understood to further strengthen CTAs into musical actions and understandings that are more relevant in cultural contexts.
3. *The organizational conduct* emerges when the child discovers the enjoyment of applying rules to her own musical games (this corresponds to Piaget's *game of rules*). These rules also play a crucial role in practices such as musical analysis and composition, where the agent develops and employs a particular and/or personal strategy to achieve the desired goal. In this sense, even using *chance* as a compositional methodology is still a choice, a self-imposed rule^10^. This phase, thus, may be thought of as moving beyond given musical cultural norms, enhancing creativity and curiosity to further understand—and *explore*—theoretical and analytical musical possibilities.

Any pedagogical work that aims to develop musical expertise, Delalande suggest, should focus on these three interactive and sensorimotor conducts (see also Nicolopoulou, 1993). But how could a music teacher or a caregiver help the child do that? Let us focus on the *explorative conduct*—which, as we argue, involves the gradual transformation of OTAs into CTAs. Here the infant comes to understand the relationship between the physical properties of the explored object and its practical possibilities for (musical) action. Research by Bondioli (1996) points to three ways in which such processes may be nurtured in children. In particular she refers to (i) *mirroring*, where the adult reproduces the spontaneous sound-based discoveries of the child; (ii) *modeling*, where the adult helps the infant to reach a musical-directed goal; and (iii) *scaffolding*, which is based on the active interaction between the two in order to develop musical ideas (here readers may also consider Gal'perin, 1957, 1967, 1969, 1982, 1989a,b,c, 1992; Vygotsky, 1978; Kozulin et al., 2003; Schiavio and Cummins, 2015).

Another important idea of Delalande concerns the continuous employment and development of the same exploratory, expressive, and organizational dynamics throughout one's musical life. In other words, earlier modes of engagement and conduct are *not* understood to be progressively replaced by those that emerge later on. Rather, primary modes continue to be crucial to creative musical engagements. Thus, improvisation and compositional practices can be seen as continuous with all of the ontogenetic processes of exploration just described, where the sounding object explored is the relevant musical instrument(s) (including the voice), and its harmonic, melodic, and timbral possibilities, which may then be organized in new ways (Menin and Schiavio, 2012). Thus, even in older children and adults, the creative enactment of new patterns and relationships necessarily involves reengagement with fundamental musical processes, including the primary body movements associated with the emergence of OTAs^11^.

## From protomusicality to teleomusicality

Thus far we have begun to lay the groundwork for an approach to musical development that is rooted in exploratory behaviors and motor activity more generally. In doing so we have attempted to show how early musicality may be conceived of as a dynamic and self-organizing phenomenon—one that cannot be fully captured in terms of developmental programs acting in response to environmental stimuli. Instead, we have suggested that the emergence of musicality in infants is better understood in terms of ongoing loops of perceptually guided action by which new affordances for behavior (or “conduct”) emerge and develop. Importantly, such processes are grounded in a primordial proclivity for movement and exploration (which result in the enactment of action understandings facilitated by the neural mirror mechanisms discussed above).

This view echoes a body of work that conceives of human development in general as a non-linear and bidirectional process (see e.g., Gibson, 1958; Thelen and Smith, 1994; Bernstein, 1996), highlighting the role of “particular experiences as catalysts for developmental cascades” (Walle, 2016; see also Spencer et al., 2009). A way to examine how motor development and different cognitive functions influence each other (see Campos et al., 2000; Iverson, 2010) might involve the adoption of dynamic systems theory (DST). Although, a full discussion on DST clearly exceeds the aims of the present contribution, it is important to introduce its main features. Very briefly, DST is a branch of mathematics that studies how complex systems evolve over time (Beer, 1995). From the molecular to the cultural level, development is examined in terms of the multiple interactions of constantly evolving sub-systems that unfold over timescales from milliseconds to years (see Kelso, 1995). Producing sounds, for example, involves coordination of limbs, activation of muscles, and, in some cases, the perception of the auditory feedback. All parts must work together to produce a successful action and its related sound. A DST perspective, thus, does not try to understand musical behaviors (in this case, sound-producing actions) by analysing each part separately. Rather, it investigates how the subsystems influence each other over time to produce a meaningful pattern that results in successful (teleomusical) actions. Such processes can be expressed via differential equations,^12^ allowing the researcher to consider the sub-systems involved as continuous and mutually interacting, rather than as discreet. Musical development, therefore, does not “depend” solely on brain maturation, nor only on the ability to master a certain perceptual or motor ability. Rather, as critical changes in each sub-system might cause a shift in the whole network, it depends on how the entire system coordinates successfully to achieve a particular (musical) goal. In line with our comments above, it should also be noted that while many approaches to early musical development rightly stress the importance of the infant's relationship with primary caregivers, our view attempts to give equal weight to the (more-or-less) independent exploratory activities of the infant. As we have anticipated, this may help to make a clearer distinction between actions and relationships that are explicitly musical and those that might best be referred to as “protomusical.” While repertoires of shared gestures and sounds enacted by infants and caregivers to communicate emotional states and bodily needs do involve parameters that are employed in musical dynamics (intensity, duration, rhythm, timbre, pitch, phrasing, and so forth), we should be careful not to simply assume that all of this is indicative of an *innate* musicality (see Trevarthen, 1997, 1999, 2001). Indeed, it may be argued that we can only define such behaviors as musical *a-posteriori*: from the immediate perspective of the infant, they arguably lack any direct relationship with “music” as such. As we just suggested, they are likely to be more indicative of non-musical goals (e.g., those related to nutrition and well-being more generally). In other words, although the earliest caregiver-infant interactions involve intersubjective motor behaviors that engage many *music-like* components, we suggest that the realization of *musicality proper* should reflect activity that is directed toward more distinctly *musical goals*. To be clear, this distinction need not undermine claims that the social and emotional aspects of our musicality can be traced in large part to such primordial infant-caregiver interactions—it is not intended to impose a discontinuity. Rather, it simply seeks to refine our understanding by considering how an explicitly *musical* behavior emerges as the infant explores its environment and begins to focus on the production of sound itself^13^.

**Figure 1:**
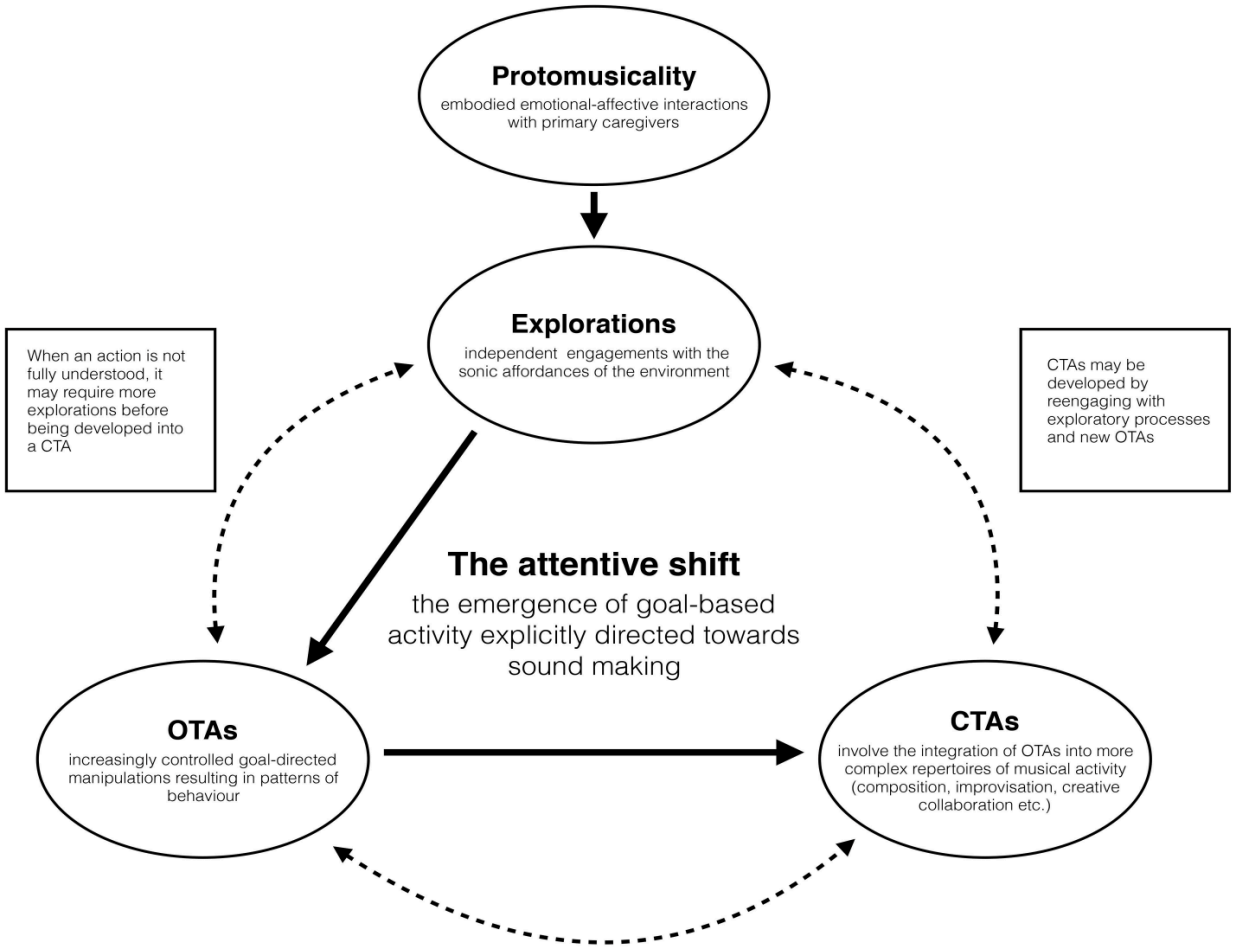
This model captures the constitution of teleomusicality through the development of OTAs into CTAs. This occurs thanks to the attentive shift that emerges through the manual exploration of the environment's sonic properties. The dotted lines show how, once the attentive shift has occurred, infants may re-engage in explorations or OTAs before developing new CTAs (adapted from Schiavio, 2014).

As we have seen, before the attentive shift, the infant's environment contains a number of possibilities for sound-making, which may be facilitated by, but not wholly dependent on, the caregiver. The caregiver might place a rattle in the infant's hand, for example, or the infant may engage with the object directly. However, as we also considered, in the first months the infant's attention is spread across the modalities, often with more focus on the kinematic aspects of the engagement (movement). Importantly, the attentive shift that occurs after 6 months involves a new, more focussed, kind of musical activity, which may now be directed toward the *sonic* possibilities of the object at hand. This occurs first through the exploration of the relationship between spontaneous movements and sound, and then via increasingly controlled goal-directed manipulations resulting in patterns of behavior (which we refer to as OTAs). It is here, we suggest, that (teleo)*musicality* as such begins (see Figure 1).

Again, this does not mean that caregivers are not involved in such processes. They (and teachers, and peers) will play an important role later on in fostering more complex and cooperative musical activities associated with the development of CTAs and Delalande's modes of conduct discussed above. However, because this perspective gives more attention to the exploratory capacities of young infants, it may offer a more nuanced approach than frameworks that focus mostly on infant-caregiver interactions. In brief, we suggest that the behavior that occurs before the attentive shift (before 6 months of age and the emergence of OTAs) is best referred to as *protomusical* because it may be directed only *inferentially* to a musically relevant goal (see also Miller, 2000; Fitch, 2005; Figure 1).

The ideas of protomusicality (which may be seen in the interaction with caregivers) and teleomusicality (which involves the development of OTAs and CTAs) may help explain the different ontogenetic trajectories by which basic musical skills emerge in infancy—e.g., the ability to synchronize with music, manipulate sound making objects toward musical goals, be sensitive to and participate in the different nuances of musical events, and to understand the musical actions of others. Likewise, this framework may also help us better understand the processes by which musical agents develop such skills beyond infancy (i.e., by constantly implementing and improving their repertoire of CTAs in new, creative ways). It could also be argued that an infant who does not transform OTAs in CTAs (perhaps because her musical environment is not *affordative* enough, or simply because her attention is captured more by the visual nuances of an object) will still possess the basic music-related actions she once developed during her exploratory sonic behaviors. Indeed, the ability to shake a rattle and listen to its sounds, to push the keys of a piano in various patterns, to move to a rhythm or sound, or beat a stick against a toy drum all afford the development of basic forms of rhythmic, melodic, and other sonic patterns. Likewise, elementary forms of dancing, singing along, humming, gesturing, and a general sense of “feeling” the music all have roots in early sensorimotor development and exploratory behaviors.

These are examples of music-like activities that make up a substrate of goal-directed musical actions that are shared among all human beings, but that develop in various ways depending on their history of interactivity with a given milieu. This can help to explain, for example, why the ability to “move to the beat,” is so widespread and easy to achieve even by non-musicians—who may nevertheless possess considerable specialized sensorimotor knowledge associated with the manifestations of music they inevitably engage with in their day to day lives as cultural beings. With this in mind, we now turn to consider the 4E framework mentioned above to offer further support for the dynamic and self-organizing approach to musical development we have been advocating for. In doing so, we will explain how this perspective aligns with our discussion of teleomusicality.

## Teleomusicality meets 4E cognition: a guide to discovery

*Teleomusicality* is a way to interpret the ontogenetic origins of human musicality as a dynamical, creative, explorative, and action-based phenomenon. It is rooted in a non-dualistic view that considers experience and behavior as continuous with each other, both being emergent properties of the ongoing interactivity occurring between a living system (brain and body) and its (social, historical, cultural, and physical) environment. Teleomusicality thus conceives of “music” as a property of such interaction, which depends upon the biological complexity of the living system (e.g., its capacity to move and meaningfully explore the world) and its history of structural coupling with its contingent niche (which provides an adequate motor repertoire). Our perspective has focussed so far on what allows this property to emerge in infancy—namely, the attentive shift that infants display between 6 and 10 months of age. To better support such claims we now frame our view within the 4E approach to cognition we began to consider early on. In doing so we will explore different theoretical accounts that point to a new understanding of human cognition (seen as *Embodied, Embedded, Enacted*, and *Extended*). Before examining in more detail what each instantiation of these “Es” entails, let us introduce some of the general tenets of the model with a brief historical excursus.

The last 60 years of research in the science of mind witnessed a number of paradigm shifts that have kept the field alive and fascinating. A major one began in the late Fifties and early Sixties, when the rise of so-called “cognitivism” challenged the dominant behaviorist view (Fodor, 1968). Behaviorism tried to shed light on the nature of cognition by developing law-like relationships between sensory inputs and behavioral outputs. However, it “allowed no reference to the internal states of the organism [and] explanations of behavior had to be formulated in terms of sensory stimuli and behavioral conditioning” (Thompson, 2007, p. 4; see also Watson, 1913; Skinner, 1938 for primary sources). The cognitivist paradigm, by contrast, offered a way to understand the inner workings of the mind in terms of a device that manipulates symbols. As such, “it is thus concerned with the formal rules and processes by which the symbols appropriately represent the world” (Thelen, 2000, p.4; see also Fodor, 1975, 1983 for primary sources). The recently emerged 4E approach to mind offers a radically different perspective. It holds that mental activity is not realized by symbolic manipulation (or information-processing) in the head. Rather, it consists of patterns of perception and action that are continuously implemented by the entire organism (brain and body), in a constant interplay with the world. Let us now consider the implications of this orientation in more detail, with a special focus on musical development.

*Embodiment* is the idea that the entire body of the living system participates in driving cognitive processes. This involves the continuous integration of sensorimotor activity (action-as-perception). The circularity between sounds and actions described in the first sections of the paper captures this idea within the domain of musical development. As we considered, developing music-related motor skills is not a property inherent to our brain alone (e.g., in terms of information stored in the head). Rather, it emerges only in relation to a given operative network. This is constituted by (i) adequate environmental supports for musical activities to take place; (ii) the living body, which adapts to the changing demands of the environment by enacting sensorimotor loops of action and perception; and (iii) the brain, which negotiates between internal and external feedbacks by integrating relevant information (e.g., via the mirror mechanism).

Importantly, the interactions between body, brain, and world strongly constrain the possibilities of the living system to act and cognize. Therefore, in addition to being embodied, the mind is also *Embedded*—it cannot be fully understood without considering how the social, physical, and cultural environment, co-constitutes one's mental life. Consider here the issue of perceptual learning, as discussed by Clarke (2005) in the context of ecological psychology. While more traditional approaches advocate for a notion of perceptual and cognitive development in terms of collection of environmental information—and the acquisition of increased “coding power” to process such information—an ecological view offers a radically different perspective (Schiavio, 2016). By this light, perception is not primarily for the accumulation of information. Rather, as we discussed above, it is for the guidance of action. In brief, this approach embraces the “profoundly active” ways living organisms shape relevant skills in relation to the environments they inhabit—a process that may occur without any “explicit *training* involved”:

[o]n first encountering a xylophone, the child's more-or-less unregulated experiments with hands or sticks will result in all kinds of accidental sounds. With unsupervised investigation, the child may discover that different kind of actions […] give rise to differentiated results […], and even that these distinctions can themselves be used to achieve other goals (Clarke, 2005, p. 23).

This resonates with the central point of the *Enactive* understanding of mind. As McGann (2014) notes, the enactive psychology is surprisingly similar with John Dewey's positions. Dewey (1896) argues against a static stimulus-response structure governing behavior, because a “stimulus” always involves a *perceptual encounter* that cannot be abstracted from the active and contingent behavioral states of the agent. Similarly, from the enactive perspective perception and cognition are seen as relational properties that emerge from the constant interplay between organisms and the worlds they participate in shaping (Varela et al., 1991). Doing music, or learning music, are two examples of possible interactivities. Music, as we mentioned in the section From Action to Musical Experience, might be conceived of as a form of embodied action humans adopt to make sense of certain properties of the environment that are discovered in early infancy. But if engaging in musical activities is best understood as the enactment of certain “points of view,” or dispositions that an agent adopts to interact with its contingent niche (including other agents), then we may conclude that (e.g., musical) “[d]evelopment, and therefore learning, is essentially an endogenously self-generating process; it is therefore unnecessary—and impossible—to ‘instruct’ it from the outside. This runs directly counter to the widespread notion that ‘learning’ is a process of ‘instruction,’ involving a process of information transfer from teacher to pupil” (Stewart et al., 2010, p. 9; see also Schiavio and Timmers, 2016; van der Schyff et al., 2016). In other words, our Elysa enacts her own world of meaning by engaging with music-like activities without being instructed to do this. It is her biological complexity, her developing physical possibilities and proclivities, her history of coupling with the surrounding (physical, social, and cultural) environment that affords possibilities, and informs her understanding of sounds, actions, interactions, and play in an ongoing way (Schiavio et al., 2016).

Finally, we could also argue that teleomusicality is an *Extended* phenomenon because it emerges in relation with devices and environments that co-constitute music-like behaviors (and not only “afford” them). Consider here the classic example from Vygotskij (reported in Borgo, 2007), where he describes how children cannot tell what they are drawing before they end their activity. In a sense, the pen and the paper shape their cognitive intuition and creativity, driving their ability to draw and elaborate conceptually on what they are engaged in. It should be noted here that such idea is not the same as the one endorsed by scholars working within classic ecological frameworks (see Gibson, 1977). The central point of the “extended mind thesis” (Clark and Chalmers, 1998) is that non-biological devices, if successfully coupled with a cognitive system, could become part of the organism's mental machinery. In a musical domain, this may resonate with the idea that the meanings enacted in early musical explorations are distributed across objects (and other agents) beyond the individual. Elysa's musical engagement with the toy rattle is not fully realized in the head, nor is it only occurring because of her ability to “pick up” information from the environment and “tune herself” to it (as in classical ecological frameworks). In a sense, rather, the mental processes that participate in Elysa's musical behaviors “extend” beyond her skin, allowing the (possible) success of her teleomusicality (i.e., non-violation of expected musical goals). Music-making, from this perspective thus becomes a pole of action and perception that extends across multiple domains, and includes surfaces, toys, sticks, and many other valuable tools (and interacting participants), which constantly inspire and drive the desire to further explore the environment and make sounds meaningfully. This has strong implications for early music pedagogy, and for research investigating the development of perception and cognition in infancy more generally.

## Conclusion

In this paper we introduced the notion of *teleomusicality* to highlight the active, self-organizing, embodied, goal-directed, and creative nature of musical development. We believe this concept may offer greater explanatory power when compared to more traditional understandings of what the early development of human musicality entails. We started our discussion with a detailed description of the mutuality of perception and action from a developmental perspective. In line with current trends in developmental cognitive neuroscience we argued for the fundamental importance active experience has for shaping the (musical) mind. In particular, we aimed to emphasize how embodied actions play a fundamental role in allowing a living system to interact with its social and physical environment in *successful* ways. One of the emergent properties of such interactions, as we discussed in the following section, is a particular kind of behavior that we can associate with what we consider “music.” Indeed, through the manual explorations of the environment that characterize the infant's first months of life, a number of “sonic discoveries” are made that stimulate the infant to further engage in activities that are explicitly directed toward sound-making. Such sonic possibilities are afforded by the environment via self-organized trajectories of goal-directed activities that become reinforced and richer over time should the infant choose to engage with them. We labeled these as *teleomusical acts*. We then individuated two sets of musically relevant actions: *original teleomusical acts* (OTA) and *constituted teleomusical acts* (CTA). While OTAs can be easily witnessed in infants' early exploratory behaviors, CTAs reflect the development and mastery of the specific goal-directed chains of action.

When an infant explores the environment and her attention is captured by the sound properties of an object, she might employ a specific goal-directed motor behavior to generate (and to modify) sounds. She can, for example, continuously hit an object in order to play with the sounds and even generate basic rhythmic, dynamic, or melodic variations. When this kind of activity exhibits clear goal-directedness (that is, when the infant is employing specific actions in order to generate and play with sounds), then the actions used by the infant can be named *original teleomusical acts*. As the infant grows older and the ability to master these actions improves, she could perform more sophisticated patterns of goal-directed sensorimotor activity, perhaps associating different OTAs without compromising the fluidity of her movements. This is evident in adult musicians, who can easily display non-associative motor behaviors while playing. When, for example, a professional guitarist explores the dynamic possibilities of the instrument to increase the tension of a given theme, she will not focus on the position of each finger independently. Her motor expertise, rather, allows her to intervene and modify the piece's dynamics through a series of coordinated movements, without unnecessary propositional thoughts. Similarly, as an infant acquires more motor expertise, she develops greater ability to reach particular musical goals with sound-making objects without focusing on single movements. Although at the beginning she might encounter difficulties and therefore focus on single actions (e.g., holding a drumstick securely, exploring the areas on the striking surface to generate different sounds), after some time she will be able to construe and perform basic sets of actions with greater accuracy and fluidity, individuating and realizing specifically musical goals (the goal of the set of action is “playing with sounds” rather than “playing with the stick”). Both OTAs and CTAs, therefore, are constituent of human musical development as they shape the degree of meaning attribution in an ongoing way (affordance, skill, creative-expressive possibilities, understanding the musical actions of others). And, as we have been considering all along, this all reflects the circular interplay between perception and action that is central to current research in neuroscience and development among others.

Moreover, we also associated such ideas with the three modes of “conduct,” proposed by Delalande. We suggested that the *explorative* conduct describes the development of OTAs into CTAs, while the *expressive* conduct reflects the early attribution of meanings to music activities—which may involve extra-musical and cultural values. We finally briefly mentioned the *organizational* conduct—where the child starts applying rules to his or her own musical game, enhancing creativity, and finding new possibilities for further interactions (which might be developed later on in analytical and compositional skills). The notion of teleomusicality was also discussed within the context of the 4E approach to cognition. Here we noted how the 4E model is well suited to capture the main aspects of teleomusicality, displaying potential to be further implemented as a framework in educational settings (see van der Schyff, 2015). In particular, we emphasized how an understanding of the ontogenetic emergence of musicality in terms of explorations and active sensorimotor loops may help us decenter the traditional focus on brain and cognitive processes, toward a more dynamical framework, where meaning-making involves the entire body of the agent in its active coupling with the world (see Schiavio and van der Schyff, 2016).

To further clarify the connection between the 4E approach and the notion of teleomusicality, consider the three components that need to be into place for *teleomusicality* to occur, specifically, (i) the skills to perform and understand (e.g., via mirror neurons) goal-directed actions, (ii) the motivation to engage in sound-oriented activities, and (iii) the ability to identify and interact with musically-relevant affordative structures in the social and/or physical environment, where the motivated behavioral patterns directed toward sounds could be enacted upon. These categories reflect three of the main central points of the 4E approach to cognition, namely the *importance of the bodily power of action*, (Embodiment), the *self-organizing properties of the living system*, who brings forth a personal point of view to meaningfully engage in motivated activities in light of the shifting demands of the environment (Enaction), and the *structural features of an agent's milieu*, which directly affects his or her intrinsic motivations and behaviors (Embedment), allowing some cognitive tasks to be offloaded in certain tools and devices of the agent's niche (Externalism). It should be noted that also in the *protomusical* phase, infants could explore the sound properties of the environment. We have argued, however, that this might reflect a *differently motivated* engagement with such activities. This does not mean that protomusical behaviors do not play a role in the flourishing of one's musical skills. In fact, mother-infant interactive behaviors do help infants develop perceptual and motor capacities that will certainly affect the organism's musical trajectories. However, we maintain that it might more helpful (in terms of clarity) to identify *musicality proper* with the intrinsically motivated sound-making activities that characterize the (sometimes unsupervised) exploratory behaviors infants engage with in their everyday life. Such activities will also have an important impact on the young organism's motor and perceptual development, and are thus internalized by the infant. The studies on mirror neurons we reviewed support this last comment by showing that goal-directed actions are mirrored when these are present in one's motor repertoire—so that before actually engaging with musically-relevant actions, infants may not posses the motor knowledge required to specifically focus on “music.” As we suggested above, this ability develops in a bidirectional way with the infants' drive to explore their environment manually. The more they explore the environment, the more they discover sound-related properties inherent to it and the actions required to produce and manipulate such properties in various ways. And as they gain such sensorimotor knowledge, they may also further explore their environment. We expect that future studies on the development of musical expertise will thus consider in greater detail the self-organizing and embodied properties inherent to such coupling, without positing too strict dichotomies between neural, behavioral, and ecological levels.

## Author contributions

AS developed the main idea and wrote the first draft. DvdS, SK-W, and RT contributed to the manuscript with comments and ideas that improved the first draft. AS, DvdS, SK-W, and RT proof-read the final version of the paper.

### Conflict of interest statement

The authors declare that the research was conducted in the absence of any commercial or financial relationships that could be construed as a potential conflict of interest.

## Footnotes

^1^This echoes Piaget's (1952) notion of *circular reactions*. This describes how—after an initial postnatal phase characterized by innate reflex actions in response to external stimulation—infants begin (between 1 and 4 months) to engage in repetitive actions centerd on exploring their body (e.g., kicking their legs). As Piaget observed, between 4 and 8 months such actions start to involve objects of the environment (e.g., shaking a rattle). This so-called stage of “secondary circular reaction” is then followed by a phase where infants become able to use their motor knowledge to reach for goals. In brief, Piaget's work highlights the tight link between perceptual and motor activities in infant development, and how they influence each other in a circular way as the infant explores the environment in increasingly complex ways.

^2^The term “teleomusicality” was originally coined by Schiavio and Menin (2011).

^3^This refers to two or more sounds played simultaneously.

^4^Here readers may consider how canonical neurons provide supporting evidence for the notion of “affordance”—a key aspect of Gibson's ecological psychology (see Gibson, 1979). This concept refers to the motor possibilities an object in the environment offers to an individual in relation to its physical capabilities—this can change through growth and experience (i.e., learning) (see Raos et al., 2006; Maranesi et al., 2014).

^5^In Rizzolatti and Luppino's words: “This motor vocabulary is constituted of ‘words,’ each of which is represented by a population of F5 neurons. Some words code the general goal of an action (e.g. grasping, holding). Others code how, within a general goal, a specific action must be executed. These words select specific ‘motor prototypes’ such as, for example, the configuration of fingers necessary for the precision grip. Finally, other words specify the temporal aspects of the action to be executed (e.g., opening of the hand). Thus, each action is represented by specific populations of neurons at different degrees of abstraction” (Rizzolatti and Luppino, 2001, p. 891).

^6^Our tentative definition of music is not meant to imply that intersubjective factors do not play a role in the development of musical abilities in infants. There is indeed a large body of work that shows how interactions between infants and caregivers shape their perceptual and motor abilities (Custodero, 2005), beginning in utero and immediately after birth (Parncutt, 2009). However, in this paper we have chosen to focus mainly on self-motivated explorations, where little or no intervention is needed. This makes musical goal-directedness simpler to identify.

^7^With regard to this point one could argue that some music-related actions do in fact involve other goals that go beyond the sole production of sounds—like *dancing* for example. However, we could say that an activity like dancing is usually enacted in a musical context, showing that the goal of similar actions remains intrinsically musical. As we will see, this is different from activities that we could define as “musical” only a-posteriori, such as clapping hands for approval, for example. In such cases, the motivation behind these actions cannot be described as “musical.”

^8^A conduct is defined as a set of coordinated actions, relevant for a specific goal.

^9^See e.g., Bart, 2004; Gopnik, 2012, for recent research on Piaget's cognitive stages more generally.

^10^The reference here is for John Cage and his well-known use of *I-Ching* for composition, during the Fifties.

^11^Here readers may be interested to consider master improviser Evan Parker's thoughts on the importance of the “bio-feedback” between performer and instrument (see Borgo, 2005).

^12^These are mathematical equations that relate certain functions with their derivatives. Generally speaking, “functions” describe physical qualities, while “derivatives” express their rates of change. *Non-linear differential equations*—the ones used by DST researchers—are adopted to describe how the complex functions of dynamically interacting systems unfold diachronically. In such cases, each equation (i.e., system) contains the other within it as a variable, so a change in the state of one will necessarily entail a change in the other. Because the functions of such systems influence each other recursively, their derivatives cannot be expressed as directly proportional to input as they can with simpler linear systems. Accordingly, such systems (e.g., organism and environment) are said to be coupled *non-linearly*. As such, they cannot be understood as separate domains, but rather as aspects of “one nondecomposable system” that evolves over time (Chemero, 2009, p. 26).

^13^These processes develop in conjunction with the (extra-musical) emotional and social experiences in order to imbue music with its broad meaning for human life. This of course is relevant to the *expressive mode of conduct* discussed by Delalande.

## References

[B1] Addessi A. R. (2008a). The musical dimension of the daily routines with under-four children, in Proceedings of the 10th International Conference on Music Perception and Cognition, eds Miyazaki K. Adachi M. Hiraga Y. Nakajima Y. Tsuzaki M. (Sapporo).

[B2] Addessi A. R. (2008b). The musical dimension of the daily routines with under-four children: changing the diaper, before sleeping, the lunch, free-play, in Conference Proceedings, Musical Development and Learning 2nd European Conference on Developmental Psychology of Music, eds Daubney A. Longhi E. Lamont A. Hargreaves D. (London: Roehampton University).

[B3] Addessi A. R. (2012). From Eco to the mirror neurons: Founding a systematic perspective of the reflexive interaction paradigm, in Proceedings of the 12nd International Conference on Music Perception and Cognition and 7th Conference of the European Society for the Cognitive Sciences of Music, eds Cambouropoulos E. Tsougras C. Mavromatis P. Pastiadis K. (Thessalonikki: School of Music Studies, University of Thessaloniki), 9–19.

[B4] Addessi A. R. (2014). Developing a theoretical foundation for the reflexive interaction paradigm with implications for training music skill and creativity. Psychomusicology 24, 214–230. 10.1037/pmu0000055

[B5] Addessi A. R. (ed.). (2015). La Creatività Musicale e Motoria dei Bambini in Ambienti Riflessivi: Proposte Didattiche con la Piattaforma MIROR. Bologna: Bononia University Press.

[B6] Addessi A. R. MAffioli M. Anelli F. (2015). The MIROR platform for young children's music and dance creativity. Reflexive interaction meets body-gesture, embodied cognition, and Laban educational dance. Perspectives 10, 9–18.

[B7] Ambrosini E. Reddy V. de Looper A. Costantini M. Lopez B. Sinigaglia C. (2013). Looking ahead: anticipatory gaze and motor ability in infancy. PLoS ONE 8:e67916. 10.1371/journal.pone.0067916 23861832 PMC3701628

[B8] Bangert M. Altenmüller E. (2003). Mapping perception to action in piano practice: a longitudinal DC-EEG study. BMC Neurosci. 4:26. 10.1186/1471-2202-4-26 14575529 PMC270043

[B9] Bart W. (2004). A commentary on D.H. Feldman's essay on Piaget's stages. N. Ideas Psychol. 22, 233–237. 10.1016/j.newideapsych.2004.12.001

[B10] Beer R. D. (1995). A dynamical systems perspective on agent-environment interaction. Artif. Intell. 72, 173–215. 10.1016/0004-3702(94)00005-L

[B11] Bernstein N. A. (1996). On dexterity and its development, in Dexterity and Its Development, eds Latash M. L. Turvey M. T. (Hillsdale, NJ: Lawrence Erlbaum Associates), 1–235.

[B12] Bertenthal B. I. Campos J. J. (1987). New directions in the study of early experience. Child Dev. 58, 560–567. 10.2307/1130198 3608640

[B13] Bondioli A. (1996). Gioco ed Educazione. Milano: Franco Angeli.

[B14] Borgo D. (2005). Sync or Swarm: Improvising Music in a Complex Age. New York, NY: Continuum.

[B15] Borgo D. (2007). Free jazz in the classroom: an ecological approach to music education. Jazz Perspect. 1, 61–88. 10.1080/17494060601061030

[B16] Bredberg G. (1968). Cellular pattern and nerve supply of the human organ of Corti. Acta Otolaryngol. Suppl. 236, 1–135. 4886545

[B17] Buccino G. Lui F. Canessa N. Patteri I. Lagravinese G. Benuzzi F. (2004). Neural circuits involved in the recognition of actions performed by non con-specifics: an fMRI study. J. Cogn. Neurosci. 16, 114–126. 10.1162/089892904322755601 15006041

[B18] Campos J. J. Anderson D. I. Barbu-Roth M. A. Hubbard E. M. Hertenstein M. J. J. Witherington D. (2000). Travel broadens the mind. Infancy 1, 149–219. 10.1207/S15327078IN0102_1 32680291

[B19] Cannon E. N. Simpson E. A. Fox N. A. Vanderwert R. E. Woodward A. L. Ferrari P. F. (2016). Relations between infants' emerging reach-grasp competence and event-related desynchronization in EEG. Dev. Sci. 19, 50–62. 10.1111/desc.12295 25754667 PMC7470427

[B20] Cannon E. N. Woodward A. L. Gredebäck G. von Hofsten C. Turek C. (2012). Action production influences 12-month-old infants' attention to others' actions. Dev. Sci. 15, 35–42. 10.1111/j.1467-7687.2011.01095.x 22251290 PMC3261504

[B21] Chemero A. (2009). Radical Embodied Cognitive Science. Cambridge, MA: MIT Press.

[B22] Chion M. (1983). Guide des Objets Sonores. Paris: Editions Buchet/Chastel.

[B23] Chion M. (1988). Du son à la chose. Analyse Musicale 11, 52–58.

[B24] Clark A. Chalmers D. (1998). The extended mind. Analyses 58, 7–19. 10.1093/analys/58.1.7

[B25] Clarke E. (2005). Ways of Listening: An Ecological Approach to the Perception of Musical Meaning. Oxford: Oxford University Press.

[B26] Cochin S. Barthelemy C. Lejeune B. Roux S. Martineau J. (1998). Perception of motion and qEEG activity in human adults. Electroencephalogr. Clin. Neurophysiol. 107, 287–295. 10.1016/S0013-4694(98)00071-6 9872446

[B27] Cochin S. Barthelemy C. Roux S. Martineau J. (1999). Observation and execution of movement: similarities demonstrated by quantified electroencephalography. Eur. J. Neurosci. 11, 1839–1842. 10.1046/j.1460-9568.1999.00598.x 10215938

[B28] Condon W. S. Sander L. W. (1974). Neonate movement is synchronized with adult speech: interactional participation and language acquisition. Science 183, 99–101. 10.1126/science.183.4120.99 4808791

[B29] Countryman J. Gabriel M. Thompson K. (2016). Children's Spontaneous vocalizations during play: aesthetic dimensions. Music Educ. Res. 18, 1–19. 10.1080/14613808.2015.1019440

[B30] Craighero L. Leo I. Umilta C. Simion F. (2011). Newborns' preference for goal-directed actions. Cognition 20, 26–32. 10.1016/j.cognition.2011.02.011 21388616

[B31] Cross I. Morley I. (2009). Music in evolution and evolutionary theory: the nature of the evidence, in Communicative Musicality, Exploring the Basis of Human Companionship, eds Malloch S. Trevarthen C. (Oxford: Oxford University Press), 61–81.

[B32] Custodero L. (2005). “Being with”: the resonant legacy of childhood's creative aesthetic, J. Aesthet. Educ. 39, 36–57. 10.1353/jae.2005.0015

[B33] Custodero L. A. Johnson-Green E. A. (2003). Passing the cultural torch: music experience and musical parenting of infants. J. Res. Music Educ. 51, 102–114. 10.2307/3345844

[B34] Custodero L. A. Johnson-Green E. A. (2008). Caregiving in counterpoint: reciprocal influences in the musical parenting of younger and older infants. Early Child Dev. Care 178, 15–39. 10.1080/03004430600601115

[B35] D'Ausilio A. (2007). The role of the mirror system in mapping complex sounds into actions. J. Neurosci. 27, 5847–5848. 10.1523/JNEUROSCI.0979-07.2007 17537954 PMC6672252

[B36] D'Ausilio A. (2009). Mirror-like mechanisms and music. ScientificWorldJournal 9, 1415–1422. 10.1100/tsw.2009.160 20024515 PMC5823102

[B37] D'Ausilio A. Altenmüller E. Olivetti Belardinelli M. Lotze M. (2006). Cross modal plasticity of the motor cortex while listening to a rehearsed musical piece. Eur. J. Neurosci. 24, 955–958. 10.1111/j.1460-9568.2006.04960.x 16930423

[B38] Damasio A. (2003). Looking for Spinoza. Joy, Sorrow and the Feeling Brain. Orlando FL: Harcourt.

[B39] Daum M. M. Prinz W. Aschersleben G. (2011). Perception and production of object-related grasping in 6-month-old infants. J. Exp. Child Psychol. 108, 810–818. 10.1016/j.jecp.2010.10.003 21092981

[B40] Delalande F. (1984). La Musique Est un Jeu D'enfant. Paris: Buchet/Chastel.

[B41] Delalande F. (1998). Music analysis and reception behaviours: sommeil by Pierre Henry. J. N. Music Res. 27, 13–66.

[B42] Delalande F. (ed.). (2009). La Nascita Della Musica. Esplorazioni Sonore nella Prima Infanzia. Milano: Franco Angeli.

[B43] Delalande F. (2013). Analyser la Musique, Pourquoi, Comment? Paris: Ina Editions.

[B44] Delalande F. (ed.). (2015). Naissance de la Musique. LES Explorations Sonores de la Première Enfance. Rennes: Presses Universitaires de Rennes.

[B45] Dewey J. (1896). The relfex arc concept in psychology. Psychol. Rev. 3, 357–370. 10.1037/h0070405

[B46] Di Pellegrino G. Fadiga L. Fogassi L. Gallese V. Rizzolatti G. (1992). Understanding motor events: a neurophysiological study. Exp. Brain Res. 91, 176–180. 10.1007/BF00230027 1301372

[B47] Dissanayake E. (2000). Antecedents of the temporal arts in early mother-infant interactions, in The Origins of Music, eds Wallin N. Merker B. Brown S. (Cambridge, MA: MIT Press), 389–410.

[B48] Eckerdal P. Merker B. (2009). “Music” and the “action song” in infant development: an interpretation, in Communicative Musicality: Narratives of Expressive Gesture and Being Human, eds Malloch S. Trevarthen C. (Oxford: Oxford University Press), 241–262.

[B49] Fadiga L. Fogassi L. Pavesi G. Rizzolatti G. (1995). Motor facilitation during action observation: a magnetic stimulation study. J. Neurophysiol. 73, 2608–2611. 7666169 10.1152/jn.1995.73.6.2608

[B50] Fitch W. T. (2005). Protomusic and protolanguage as alternatives to protosign (comment on Arbib). Behav. Brain Sci. 28, 132–133. 10.1017/S0140525X05290039

[B51] Fodor J. (1968). Psychological Explanation. New York, NY: Random House.

[B52] Fodor J. (1975). The Language of Thought. Cambridge, MA; London: Harvard University Press.

[B53] Fodor J. (1983). The Modularity of Mind: An Essay on Faculty Psychology. Cambridge, MA: MIT Press.

[B54] Fogassi L. Ferrari P. F. Gesierich B. Rozzi S. Chersi F. Rizzolatti G. (2005). Parietal lobe: from action organization to intention understanding. Science 308, 662–667. 10.1126/science.1106138 15860620

[B55] Fox N. A. Bakermans-Kranenburg M. J. Yoo K. H. Bowman L. C. Cannon E. N. Vanderwert R. E.. (2016). Assessing human mirror activity with EEG Mu rhythm: a meta-analysis. Psychol. Bull. 142, 291–313. 10.1037/bul0000031 26689088 PMC5110123

[B56] Gal'perin P. Ya. (1957). An experimental study in the formation of mental actions, in Psychology in the Soviet Union, ed Simon B. (London: Routledge and Kegan Paul), 213–225.

[B57] Gal'perin P. Ya. (1967). On the notion of internalization. Sov. Psychol. 5, 28–33.

[B58] Gal'perin P. Ya. (1969). Stages in the development of mental acts, in A handbook of Contemporary Soviet Psychology, eds Cole M. Maltzman I. (New York, NY: Basic Books), 249–273.

[B59] Gal'perin P. Ya. (1982). Intellectual capabilities among older preschool children: on the problem of training and development, in Review of Child Develoment Research, Vol. 6, ed Hartup W. W. (Chicago, IL: University of Chicago Press), 526–546.

[B60] Gal'perin P. Ya. (1989a). Study of the intellectual development of the child. Sov. Psycho. 27, 26–44.

[B61] Gal'perin P. Ya. (1989b). Mental actions as a basis for the formation of thoughts and images. Sov. Psychol. 27, 45–64.

[B62] Gal'perin P. Ya. (1989c). Organization of mental activity and the effectiveness of learning. Sov. Psychol. 27, 65–82.

[B63] Gal'perin P. Ya. (1992). Stage-by-stage formation as a method of psychological investigation. J. Rus. East Eur. Psychol. 30, 60–80.

[B64] Gallese V. (2005). Embodied simulation: from neurons to phenomenal experience. Phenomenon Cogn. Sci. 4, 23–48. 10.1007/s11097-005-4737-z

[B65] Gallese V. Fadiga L. Fogassi L. Rizzolatti G. (1996). Action recognition in the premotor cortex. Brain 119, 593–609. 10.1093/brain/119.2.593 8800951

[B66] Galloway G. C. (2005). In memoriam: esther Thelen May 20, 1941–December 29, 2004. Dev. Psychobiol. 47, 103–107. 10.1002/dev.20084 16136565

[B67] Gergely G. Ndasdy Z. Csibra G. Biró S. (1995). Taking the intentional stance at 12 months of age. Cognition 56, 165–193. 10.1016/0010-0277(95)00661-H 7554793

[B68] Gerson S. A. Bekkering H. Hunnius S. (2015a). Short-term motor training, but not observational training, alters neurocognitive mechanisms of action processing in infancy. J. Cogn. Neurosci. 27, 1207–1214. 10.1162/jocn_a_00774 25514654

[B69] Gerson S. A. Schiavio A. Timmers R. Hunnius S. (2015b). Active drumming experience increases infants' sensitivity to audiovisual synchronicity during observed drumming actions. PLoS ONE 10:e0130960 10.1371/journal.pone.0130960 26111226 PMC4482535

[B70] Gibson E. J. Walker A. S. (1984). Development of knowledge of visual-tactual affordances of substance. Child Dev. 55, 453–460. 10.2307/1129956 6723444

[B71] Gerson S. A. Woodward A. L. (2014a). The joint role of trained, untrained, and observed actions at the origins of goal recognition. Infant Behavior. Dev. 37, 94–104. 10.1016/j.infbeh.2013.12.013 24468646 PMC3951724

[B72] Gerson S. A. Woodward A. L. (2014b). Learning from their own actions: the unique effect of producing actions on infants' action understanding. Child Dev. 85, 264–277. 10.1111/cdev.12115 23647241 PMC3740060

[B73] Gibson E. J. (1988). Exploratory behavior in the development of perceiving, acting, and the acquiring of knowledge. Annu. Rev. Psychol. 39, 1–41. 10.1146/annurev.ps.39.020188.000245

[B74] Gibson J. J. (1958). Visually controlled locomotion and visual orientation in animals. Br. J. Psychol. 49, 182–194. 10.1111/j.2044-8295.1958.tb00656.x 13572790

[B75] Gibson J. J. (1977). The theory of affordances, in Perceiving, Acting and Knowing: Toward an Ecological Psychology, eds Shaw R. Bransford J. (Hillsdale, MI: Lawrence Erlbaum), 67–82.

[B76] Gibson J. J. (1979). The Ecological Approach to Visual Perception. Boston MA: Houghton Mifflin Company.

[B77] Godøy R. I. (1997). Knowledge in music theory by shapes of musical objects and sound producing actions, in Music, Gestalt and Computing. Studies in Cognitive and Systematic Musicology, ed Leman M. (Berlin; Heidelberg: Springer Verlag), 106–110.

[B78] Godøy R. I. (2003). Motor-mimetic music cognition. Leonardo 36, 317–319. 10.1162/002409403322258781

[B79] Gopnik A. (2012). Scientific thinking in young children: theoretical advances, empirical research, and policy implications. Science 337, 1623–1627. 10.1126/science.1223416 23019643

[B80] Gottlieb G. (1991a). Experiential canalization of behavioral development: results. Dev. Psychol. 27, 35–39.

[B81] Gottlieb G. (1991b). Experiential canalization of behavioral development: theory. Dev. Psychol. 27, 4–13.

[B82] Gratier M. (2003). Expressive timing and interactional synchrony between mothers and infants: cultural similarities, cultural differences, and the immigration experience. Cogn. Dev. 18, 533–554. 10.1016/j.cogdev.2003.09.009

[B83] Gratier M. (2007). Musicalité, style et appartenance, in Geste, Temps et Musicalité, eds Imberty M. Gratier M. (Paris: L'Harmattan), 69–100.

[B84] Gratier M. Trevarthen C. (2008). Musical narrative and motives for culture in mother-infant vocal interaction. J. Conscious. Stud. 15, 122–158.

[B85] Hannon E. E. Trehub S. E. (2005). Tuning in to musical rhythms: infants learn more readily than adults. Proc. Natl. Acad. Sci. U.S.A. 102, 12639–12643. 10.1073/pnas.0504254102 16105946 PMC1194930

[B86] He M. Walle E. A. Campos J. J. (2015). A cross-national investigation of the relationship between infant walking and language development. Infancy 20, 283–305. 10.1111/infa.12071

[B87] Ilari B. Moura A. Bourscheidt L. (2011). Between interactions and commodities: musical parenting of infants and toddlers in Brazil. Music Educ. Res. 13, 51–67. 10.1080/14613808.2011.553277

[B88] Imberty I. (1995). Développement linguistique et musical de l'enfant d'âge préscolaire et scolaire, in Naissance et Développement du Sens Musical, eds Deliege I. Sloboda J. (Paris: PUF), 223–250.

[B89] Imberty M. (2009). There is no musicality without intentionality. Coming back to the origins of human musicality, in MERYC2009 Proceedings of the 4th Conference of the European Network of Music Educators and Researchers of Young Children, eds Addessi A. R. Young S. (Bologna: Bononia University Press).

[B90] Iverson J. M. (2010). Developing language in a developing body: the relationship between motor development and language development. J. Child Lang. 37, 229–261. 10.1017/S0305000909990432 20096145 PMC2833284

[B91] Jaffe J. Beebe B. Feldstein S. Crown C. L. Jasnow M. (2001). Rhythms of dialogue in infancy: coordinated timing in development. Monogr. Soc. Res. Child Dev. 66, 1–132. 11428150

[B92] Kanakogi Y. Itakura S. (2011). Developmental correspondence between action prediction and motor ability in early infancy. Nat. Commun. 2:341. 10.1038/ncomms1342 21654641

[B93] Keefe D. H. Bulen J. C. Campbell S. L. Burns E. M. (1994). Pressure transfer function and absorption cross section from the diffuse field to the human infant ear canal. J. Acoust Soc. Am. 95, 355–371. 10.1121/1.408380 8120247

[B94] Kelso S. (1995). Dynamic Patterns. The Self-Organization of Brain and Behaviour. Cambridge MA: MIT Press.

[B95] Keysers C. (2007). Mirror Neurons. New Encyclopaedia of Neuroscience. Amsterdam: Elsevie Press.

[B96] Keysers C. Gazzola V. (2010). Social neuroscience: mirror neurons recorded in humans. Curr. Biol. 20, R353–R354. 10.1016/j.cub.2010.03.013 21749952

[B97] Kohler E. Keysers C. Umiltà M. A. Fogassi L. Gallese V. Rizzolatti G. (2002). Hearing sounds, understanding actions: action representation in mirror neurons. Science 297, 846–848. 10.1126/science.1070311 12161656

[B98] Kozulin A. Gindis B. Ageyev V. Miller S. (2003). Vygotsky's Educational Theory in Cultural Context. Cambridge, MA: Cambridge University Press.

[B99] Krueger J. (2013). Empathy, enaction, and shared musical experience: Evidence from infant cognition, in The Emotional Power of Music: Multidisciplinary Perspectives on Musical Arousal, Expression, and Social Control, eds Cochrane T. Fantini B. Scherer K. (Oxford: Oxford University Press), 177–196.

[B100] Lecanuet J. P. Granier-Deferre C. Busnel M. C. (1988). Fetal cardiac and motor responses to octave-band noises as a function of central frequency intensity and heart rate variability. Early Hum. Dev. 18, 81–93. 10.1016/0378-3782(88)90045-X 3224585

[B101] Lecanuet J. P. Granier-Deferre C. Busnel M. C. (1991). Prenatal Familiarization, in From Basic Langage to Discourse Bases, eds Piéraut-Le Bonniec G. Dolitsky M. (Amsterdam; Philadelphia, PA: John Benjamin Publisher Co), 31–44.

[B102] Lecanuet J. P. Granier-Deferre C. Cohen H. Le Houezec R. Busnel M. C. (1986). Fetal responses to acoustic stimulation depend on heart rate variability pattern stimulus intensity and repetition. Early Hum. Dev. 13, 269–283. 10.1016/0378-3782(86)90061-7 3720613

[B103] Leman M. (2007). Embodied Music Cognition and Mediation Technology. Cambridge: MIT Press.

[B104] Leman M. Desmet F. Styns F. Van Noorden L. Moelants D. (2009). Sharing musical expression through embodied listening: a case study based on Chinese Guqin Music. Music Percept. 26, 263–268. 10.1525/mp.2009.26.3.263

[B105] Leman M. Maes P.-J. (2015). The role of embodiment in the perception of music. Empirical Musicol. Rev. 99, 236–246. 10.18061/emr.v9i3-4.4498

[B106] Malloch S. Trevarthen C. (eds.). (2009). Communicative Musicality. Exploring the Basis of Human Companionship. Oxford: Oxford University Press.

[B107] Maranesi M. Bonini L. Fogassi L. (2014). Cortical processing of object affordances for self and others' action. Front. Psychol. 5:538. 10.3389/fpsyg.2014.00538 24987381 PMC4060298

[B108] McCall R. B. (1974). Exploratory manipulation and play in the human infant. Monogr. Soc. Res. Child Dev. 39, 1–88. 10.2307/1166007 4444723

[B109] McGann M. (2014). Enacting a social ecology: radically embodied intersubjectivity. Front. Psychol. 5:1321. 10.3389/fpsyg.2014.01321 25477844 PMC4235264

[B110] Menin D. Schiavio A. (2012). Rethinking musical affordances. AVANT. Trends Interdiscipl. Stud. 3, 202–215.

[B111] Merleau-Ponty M. (1945). Phénoménologie de la perception, Paris: Gallimard; Phenomenology of Perception, Donald Landes (trans.). London: Routledge.

[B112] Miller G. F. (2000). Evolution of human music through sexual selection, in The Origins of Music, eds Wallin N. L. Merker B. Brown S. (Cambridge MA: MIT Press), 329–360.

[B113] Molenberghs P. Cunnington R. Mattingley J. B. (2012). Brain regions with mirror properties: a meta-analysis of 125 human fMRI studies. Neurosci. Biobehav. Rev. 36, 341–349. 10.1016/j.neubiorev.2011.07.004 21782846

[B114] Moore D. R. Jeffery G. (1994). Development of auditory and visual systems in the fetus, in Textbook of Fetal Physiology, eds Thorburn G. D. Harding R. (Oxford: Oxford University Press), 278–286.

[B115] Mukamel R. Ekstrom A. D. Kaplan J. Iacoboni M. Fried I. (2010). Single-neuron responses in humans during execution and observation of actions. Curr. Biol. 20, 750–756. 10.1016/j.cub.2010.02.045 20381353 PMC2904852

[B116] Nakata T. Trehub S. E. (2004). Infants' responsiveness to maternal speech and singing. Infant Behav. Dev. 27, 455–464. 10.1016/j.infbeh.2004.03.002

[B117] Nattiez J. J. (1987). Musicologie Générale et Sémiologie. Paris: Christian Bourgois.

[B118] Nicolopoulou A. (1993). Play, cognitive development, and the social world: piaget, vygotsky, and beyond. Hum. Dev. 3, 1–23. 10.1159/000277285

[B119] Nijs L. Leman M. (2016). Improvisare con e senza la componente MIRIR-Impro della piattaforma MIROR: proposte di pratiche con i bambini secondo un approccio task-based, in La Creatività Musicale e Motoria dei Bambini in Ambienti Riflessivi : Proposte Didattiche con la Piattaforma Miror, ed Addessi A. R. (Bologna: Bononia Universita Press), 121–138.

[B120] Nyström P. (2008). The infant mirror neuron system studied with high density EEG. Soc. Neurosci. 3, 334–347. 10.1080/17470910701563665 18979389

[B121] Olsho L. W. (1984). Infant frequency discrimination. Infant Behav. Dev. 7, 27–35. 10.1016/S0163-6383(84)80020-X

[B122] Oudgenoeg-Paz O. Volman M. J. M. Leseman P. P. M. (2012). Attainment of sitting and walking predicts development of productive vocabulary between ages 16 and 28 months. Infant Behav. Dev. 35, 733–736. 10.1016/j.infbeh.2012.07.010 22982273

[B123] Overy K. Molnar-Szakacs I. (2009). Being together in time: musical experience and the mirror neuron system. Music Percept. 26, 489–504. 10.1525/mp.2009.26.5.489

[B124] Papoušek M. (1996). Intuitive parenting: a hidden source of musical stimulation in infancy, in Musical Beginnings: Origins and Development of Musical Competence, eds Deliege I. Sloboda J. (New York, NY: Oxford University Press), 88–112.

[B125] Parncutt R. (2009). Prenatal and infant conditioning, the mother schema, and the origins of music and religion. Music. Sci. 2009–2010, 119–150. 10.1177/1029864909013002071

[B126] Perani D. Saccuman M. C. Scifo P. Spada D. Andreolli G. Rovelli R.. (2010). Functional specializations for music processing in the human newborn brain. Proc. Natl. Acad. Sci. U.S.A. 107, 4758–4763. 10.1073/pnas.0909074107 20176953 PMC2842045

[B127] Perone S. Madole K. L. Ross-Sheehy S. Carey M. Oakes L. M. (2009). The relation between infants' activity with objects and attention to object appearance. Dev. Psychol. 44, 1242–1248. 10.1037/0012-1649.44.5.1242 18793058 PMC2596282

[B128] Perone S. Oakes L. M. (2006). It clicks when it is rolled and squeaks when it is squeezed: what 10-month-old infants learn about function. Child Dev. 77, 1608–1622. 10.1111/j.1467-8624.2006.00962.x 17107449

[B129] Philips-Silver J. Trainor L. J. (2007). Hearing what the body feels: auditory encoding of rhythmic movement. Cognition 105, 533–546. 10.1016/j.cognition.2006.11.006 17196580

[B130] Piaget J. (1952). The Origins of Intelligence in Children. New York, NY: International Universities Press.

[B131] Piaget J. (1964). Development and learning, in Piaget Rediscovered, eds Ripple R. Rockcastle V. (Ithaca: Cornel University), 7–20.

[B132] Pierroutsakos S. L. DeLoache J. S. (2003). Infants' manual exploration of pictorial objects varying in realism. Infancy 4, 141–156. 10.1207/S15327078IN0401_7

[B133] Raos V. Umilta M. A. Murata A. Fogassi L. Gallese V. (2006). Functional properties of grasping-related neurons in the ventral premotor area F5 of the macaque monkey. J. Neurophysiol. 95, 709–729. 10.1152/jn.00463.2005 16251265

[B134] Reybrouck M. (2005). A biosemiotic and ecological approach to music cognition: event perception between auditory listening and cognitive economy. Axiomates 15, 391–409. 10.1007/s10516-004-6679-4

[B135] Reybrouck M. (2012). Musical sense-making and the concept of affordance: an ecosemiotic and experiential approach. Biosemiotics 5, 391–409. 10.1007/s12304-012-9144-6

[B136] Rizzolatti G. Camarda R. Fogassi L. Gentilucci M. Luppino G. Matelli M. (1988). Functional organization of inferior area 6 in the macaque monkey. II. Area F5 and the control of distal movements. Exp. Brain Res. 71, 491–507. 10.1007/BF00248742 3416965

[B137] Rizzolatti G. Luppino G. (2001). The cortical motor system. Neuron 31, 889–901. 10.1016/S0896-6273(01)00423-8 11580891

[B138] Rizzolatti G. Sinigaglia C. (2008). Mirrors in the Brain. How Our Minds Share Actions and Emotions. Oxford: Oxford University Press.

[B139] Robson S. J. Kuhlmeier V. A. (2016). Infants' understanding of object-directed action: an interdisciplinary synthesis. Front. Psychol. 7:111. 10.3389/fpsyg.2016.00111 26903918 PMC4746616

[B140] Rowlands M. (2010). The New Science of the Mind. Cambridge, MA: MIT Press.

[B141] Ruff H. A. (1986). Components of attention during infants' manipulative exploration. Child Dev. 57, 105–114. 10.2307/1130642 3948587

[B142] Ruff H. A. (1984). Infants' manipulative exploration of objects: effects of age and object characteristics. Dev. Psychol. 20, 9–20.

[B143] Schaeffer P. (1966). Traité des Objets Musicaux. Paris: Editions du Seuil.

[B144] Schiavio A. (2014). Music in (en)Action. Sense-Making and Neurophenomenology of Musical Experience. Ph.D. thesis, The University of Sheffield.

[B145] Schiavio A. (2016). Enactive affordances and the interplay of biological and phenomenological subjectivity. Construct. Found. 11, 315–317.

[B146] Schiavio A. Altenmüller E. (2015). Exploring music-based rehabilitation for parkinsonism through embodied cognitive science. Front. Neurol. 6:217. 10.3389/fneur.2015.00217 26539155 PMC4609849

[B147] Schiavio A. Cummins F. (2015). An inter(en)active approach to musical agency and learning, in Proceedings of ICMEM 2015, International Conference on the Multimodal Experience of Music, eds Timmers R. Dibben N. Eitan Z. Granot R. Metcalfe T. Schiavio A. Williamson V. (Sheffield: HRI Online Publications).

[B148] Schiavio A. Menin D. (2011). Mirroring teleomusical acts, Poster Presented at the IV Neuromusic Conference (Edinburgh: University of Edinburgh).

[B149] Schiavio A. Timmers R. (2016). Motor and audiovisual learning consolidate auditory memory of tonally ambiguous melodies. Music Percept. 34, 21–32. 10.1525/mp.2016.34.1.21

[B150] Schiavio A. van der Schyff D. (2016). Beyond musical qualia. Reflecting on the concept of experience. Psychomusicol. Music Mind Brain 26, 366–378.

[B151] Schiavio A. van der Schyff D. Cespedes-Guevara J. Reybrouck M. (2016). Enacting musical emotions. Sense-making, dynamic systems, and the embodied mind. Phenomenol. Cogn. Sci. 1–25. 10.1007/s11097-016-9477-8

[B152] Sheya A. Smith L. B. (2010a). Development through sensory-motor coordinations, in Enaction: Towards a New Paradigm for Cognitive Science, eds Stewart J. Gapenne O. Di Paolo E. (Cambridge, MA: MIT Press), 123–144.

[B153] Sheya A. Smith L. B. (2010b). Changing priority maps in 12- to 18-month-olds: an emerging role for object properties. Psychol. Bull. Rev. 17, 22–28. 10.3758/PBR.17.1.22 20081156 PMC2887713

[B154] Skinner B. F. (1938). The Behaviour of Organisms: An Experimental Analysis. New York, NY: Appleton-Century-Crofts.

[B155] Smith L. Thelen E. (2003). Development as a dynamic system. Trend Cogn. Sci. 7, 343–344. 10.1016/S1364-6613(03)00156-6 12907229

[B156] Smith S. L. Gerhardt K. J. Griffiths S. K. Huang X. Abrams R. M. (2003). Intelligibility of sentences recorded from the uterus of a pregnant ewe and from the fetal inner ear. Audiol. Neurootol. 8, 347–353. 10.1159/000073519 14566105

[B157] Soley G. Hannon E. (2010). Infants prefer the musical meter of their own culture: a cross-cultural comparison. Dev. Psychol. 46, 286–292. 10.1037/a0017555 20053025

[B158] Sommerville J. A. Woodward A. L. Needham A. (2005). Action experience alters 3-month-old infants' perception of others' actions. Cognition 96 B1–B11. 10.1016/j.cognition.2004.07.004 15833301 PMC3908452

[B159] Soska K. C. Adolph K. E. Johnson S. P. (2010). Systems in development: motor skill acquisition facilitates three-dimensional object completion. Dev. Psychol. 46, 129–138. 10.1037/a0014618 20053012 PMC2805173

[B160] Spencer J. P. Blumberg M. S. McMurray B. Robinson S. R. Samuelson L. K. Tomblin J. B. (2009). Short arms and talking eggs: why we should no longer abide the nativist-empiricist debate. Child Dev. Perspect. 3, 79–87. 10.1111/j.1750-8606.2009.00081.x 19784383 PMC2750899

[B161] Spencer J. P. Clearfield M. Corbetta D. Ulrich B. Buchanan P. Schöner G. (2006). Moving toward a grand theory of development: in memory of esther thelen. Child Dev. 77, 1521–1538. 10.1111/j.1467-8624.2006.00955.x 17107442

[B162] Stern D. N. Jaffe J. Beebe B. Bennett S. L. (1975). Vocalization in unison and alternation: two modes of communication within the mother-infant dyad. Ann. N.Y. Acad. Sci. 263, 89–100. 1060437 10.1111/j.1749-6632.1975.tb41574.x

[B163] Stern D. N. Spieker S. MacKain K. (1982). Intonation contours as signals in maternal speech to prelinguistic infants. Dev. Psychol. 18, 727–735.

[B164] Stewart J. Gapenne O. Di Paolo E. (eds.). (2010). Enaction: Towards a New Paradigm for Cognitive Science. Cambridge, MA: MIT Press.

[B165] Thelen E. (1989). Self-organization in developmental processes: can systems approaches work?, in Systems and Development: The Minnesota Symposia on Child Psychology, Vol. 22, eds Gunnar M. Thelen E. (Hillsdale, MI: Erlbaum), 17–171.

[B166] Thelen E. (1994). Three-month-old infants can learn task-specific patterns of interlimb coordination. Psychol. Sci. 5, 280–285. 10.1111/j.1467-9280.1994.tb00626.x

[B167] Thelen E. (2000). Grounded in the world: developmental origins of the embodied mind. Infancy 1, 3–28. 10.1207/S15327078IN0101_02 32680313

[B168] Thelen E. Schoner G. Scheier C. Smith L. B. (2001). The dynamics of embodiment: a field theory of infant preservative reaching. Behav. Brain Sci. 24, 1–86. 10.1017/S0140525X01003910 11515285

[B169] Thelen E. Smith L. B. (1994). A Dynamic Systems Approach to the Development of Cognition and Action. Cambridge, MA: MIT Press.

[B170] Thompson E. (2007). Mind in Life: Biology, Phenomenology, and the Sciences of Mind. Cambridge; London: Harvard University Press.

[B171] Trainor L. J. Trehub S. E. (1992). A comparison of infants' and adults' sensitivity to Western musical structure. J. Exp. Psychol. Hum. Percept. Perform. 18, 394–402. 10.1037/0096-1523.18.2.394 1593226

[B172] Trehub S. E. (2003a). The developmental origins of musicality. Nat. Neurosci. 6, 669–673. 10.1038/nn1084 12830157

[B173] Trehub S. E. (2003b). Absolute and relative pitch processing in tone learning tasks. Dev. Sci. 6, 46–47. 10.1111/1467-7687.00251_1

[B174] Trehub S. E. (2003c). In the beginning, there was music. Bull. Psychol. Arts 4, 42–44.

[B175] Trehub S. E. (2003d). Musical predispositions in infancy: an update, in The Cognitive Neuroscience of Music, eds Zatorre R. Peretz I. (Oxford: Oxford, University Press), 3–20.

[B176] Trehub S. E. (2003e). Toward a developmental psychology of music. Ann. N.Y. Acad. Sci. 999, 402–413. 14681165 10.1196/annals.1284.051

[B177] Trehub S. E. (2009). Music lessons from infants, in Oxford Handbook of Music Psychology, eds Hallam S. Cross I. Thaut M. (Oxford: Oxford University Press), 229–234.

[B178] Trehub S. E. Bull D. Thorpe L. A. (1984). Infants' perception of melodies: the role of melodic contour. Child Dev. 55, 821-830. 10.2307/1130133 6734320

[B179] Trevarthen C. (1997). Empatia e Biologia. Milano: Raffello Cortina.

[B180] Trevarthen C. (1999). Musicality and the intrinsic motive pulse: evidence from human psychobiology and infant communication. Music. Sci. 3, 155–215. 10.1177/10298649000030S109

[B181] Trevarthen C. (2001). The neurobiology of early communication: intersubjective regulations in human brain development, in Handbook on Brain and Behavior in Human Development, eds Kalverboer A. F. Gramsbergen A. (Dordrecht: Kluwer), 841–882.

[B182] Trevarthen C. Malloch S. N. (2000). The dance of wellbeing: defining the musical therapeutic effect. Nordic J. Music Ther. 9, 3–17. 10.1080/08098130009477996

[B183] van der Meer A. L. H. van der Weel F. R. Lee D. N. (1995). The functional significance of arm movements in neonates. Science 267, 693–695. 10.1126/science.7839147 7839147

[B184] van der Schyff D. (2015). Music as a manifestation of life: exploring enactivism and the ‘eastern perspective’ for music education. Front. Psychol. 6:345. 10.3389/fpsyg.2015.00345 25870576 PMC4375918

[B185] van der Schyff D. Schiavio A. Elliott D. J. (2016). Critical ontology for an enactive music pedagogy. Action Critic. Theor. Music Educ. 15, 81–121. 10.22176/act15.5.81

[B186] Varela F. Thompson E. Rosch E. (1991). The Embodied Mind: Cognitive Science and Human Experience. Cambridge, MA: MIT Press.

[B187] Volpe G. Varni G. Mazzarino B. Addessi A. R. (2012). BeSound: embodied reflexion for music education in childhood, in Proceedings of the 11th International Conference on Interaction Design and Children (New York, NY: ACM), 172–175.

[B188] von Hofsten C. (1982). Eye-hand coordination in the newborn. Dev. Psychol. 18, 450–461. 10.1037/0012-1649.18.3.450

[B189] Vygotsky L. (1978). Mind in Society. The Development of Higher Psychological Processes. Cambridge, MA; London: Harvard University Press.

[B190] Walle E. A. (2016). Infant social development across the transition from crawling to walking. Front. Psychol. 7:960. 10.3389/fpsyg.2016.00960 27445923 PMC4921474

[B191] Watson J. B. (1913). Psychology as the behaviourist views it. Psychol. Rev. 20, 158–177.

[B192] Werner L. A. (2002). Infant auditory capabilities. Curr. Opin. Otolaryngol. Head Neck Surg. 10, 398–402. 10.1097/00020840-200210000-00013

[B193] Woodward A. L. (1998). Infants selectively encode the goal object of an actor's reach. Cognition 69, 1–34. 10.1016/S0010-0277(98)00058-4 9871370

[B194] Woodward A. L. (2009). Infants' grasp of others' intentions. Curr. Direct. Psychol. Sci. 18, 53–57. 10.1111/j.1467-8721.2009.01605.x 23645974 PMC3640581

[B195] Woodward A. L. Gerson S. A. (2014). Mirroring and the development of action understanding. Philos. Trans. R. Soc. Lond. B Biol. Sci. 369:20130181. 10.1098/rstb.2013.0181 24778377 PMC4006183

